# Arthroscopic Treatment of Synovial Chondromatosis in the Ankle Joint

**DOI:** 10.7759/cureus.1983

**Published:** 2017-12-23

**Authors:** Daniel R Kunzler, Pejma Shazadeh Safavi, Brittney J Warren, Cory F Janney, Vinod Panchbhavi

**Affiliations:** 1 School of Medicine, University of Texas Medical Branch at Galveston; 2 University of Texas Medical Branch at Galveston; 3 Department of Orthopedics, University of Texas Medical Branch at Galveston; 4 Department of Orthopedics, United States Navy, University of Texas Medical Branch at Galveston

**Keywords:** synovial chondromatosis, arthroscopy, ankle, synovectomy, loose bodies

## Abstract

Synovial chondromatosis of the ankle is rare and sparsely documented. Traditional surgical intervention is open loose body excision and synovectomy. Upon literature review, only two other cases were found to be managed arthroscopically. We report a case of synovial chondromatosis in a 54-year-old man leading to pain and limited range of motion of his ankle. This unique case of extensive nodule formation was treated via a three-port arthroscopic approach. Removal of loose bodies and synovectomy were successfully performed arthroscopically. A total of 76 loose bodies were removed and synovectomy performed using a 3.5 mm diameter full radius shaver. This case demonstrates that a three-port arthroscopic approach can provide adequate treatment while maintaining the superior risk profile inherent to arthroscopic intervention.

## Introduction

Synovial chondromatosis can be a chronic and debilitating disease if left untreated. Joint damage results from progressive degeneration of articular structures secondary to loose, neoplastic cartilaginous nodules in the joint space [1]. The disease presents twice as often in males versus females and predominantly affects patients in the third through fifth decades of life. The knee joint is the most commonly involved and presents in up to 60-70% of cases, followed by the hip, elbow, and shoulder. The stages of synovial chondromatosis include the early stage without loose bodies, a transitional stage with intrasynovial nodules, and the late stage with multiple intra-articular loose bodies. Synovial chondromatosis is described as primary, without identifiable pathology, or secondary, associated with pre-existing joint pathology such as osteoarthritis, inflammatory arthritis, osteonecrosis, or other intra-articular derangements. Secondary synovial chondromatosis typically has larger free bodies and more size variation in the loose bodies. Synovial chondromatosis of the ankle is rare and sparsely documented in the current literature [2, 3]. Conventional surgical intervention for loose bodies is open excision with synovectomy [4]. We present a unique case of synovial chondromatosis with extensive nodule formation treated with a three-port arthroscopic approach.

## Case presentation

A 54-year-old male patient with no significant past medical history presented to our clinic for progressive deterioration of his left ankle after sustaining a hyper-dorsiflexion injury eight months prior. He had no previous ankle injuries or surgeries and recently noted decreased range of motion and intermittent “locking up” of the ankle. The pain was described as a migrating, “pinching” sensation causing significant limitations to ambulation. Initial conservative measures including rest, physical therapy, and nonsteroidal anti-inflammatory drugs (NSAIDS), failed to provide adequate relief prior to orthopedic consultation.

**Figure 1:**
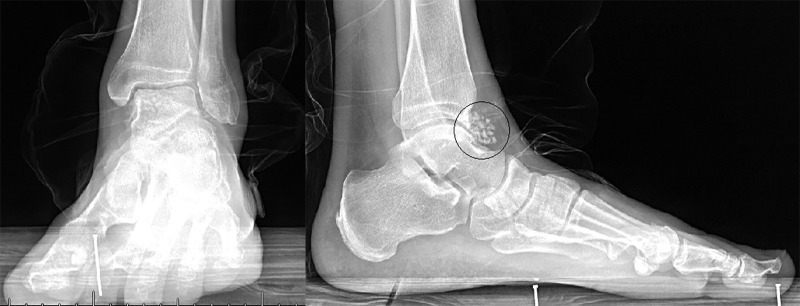
Preoperative Radiographs. Preoperative radiographs demonstrate innumerable subcentimeter loose bodies within the tibiotalar joint. Anteroposterior radiograph shown on the left, and lateral radiograph shown on the right with loose bodies outlined by black circle.

The patient presented with global swelling and tenderness to palpation around the tibiotalar joint. The range of motion on initial exam was 5° of dorsiflexion and 10° of plantar flexion with severe limitations secondary to pain. Dorsalis pedis pulse and sensation to light touch was intact. Preoperative radiographs are shown in Figure 1 which reveal innumerable subcentimeter loose bodies within the tibiotalar joint.

**Figure 2:**
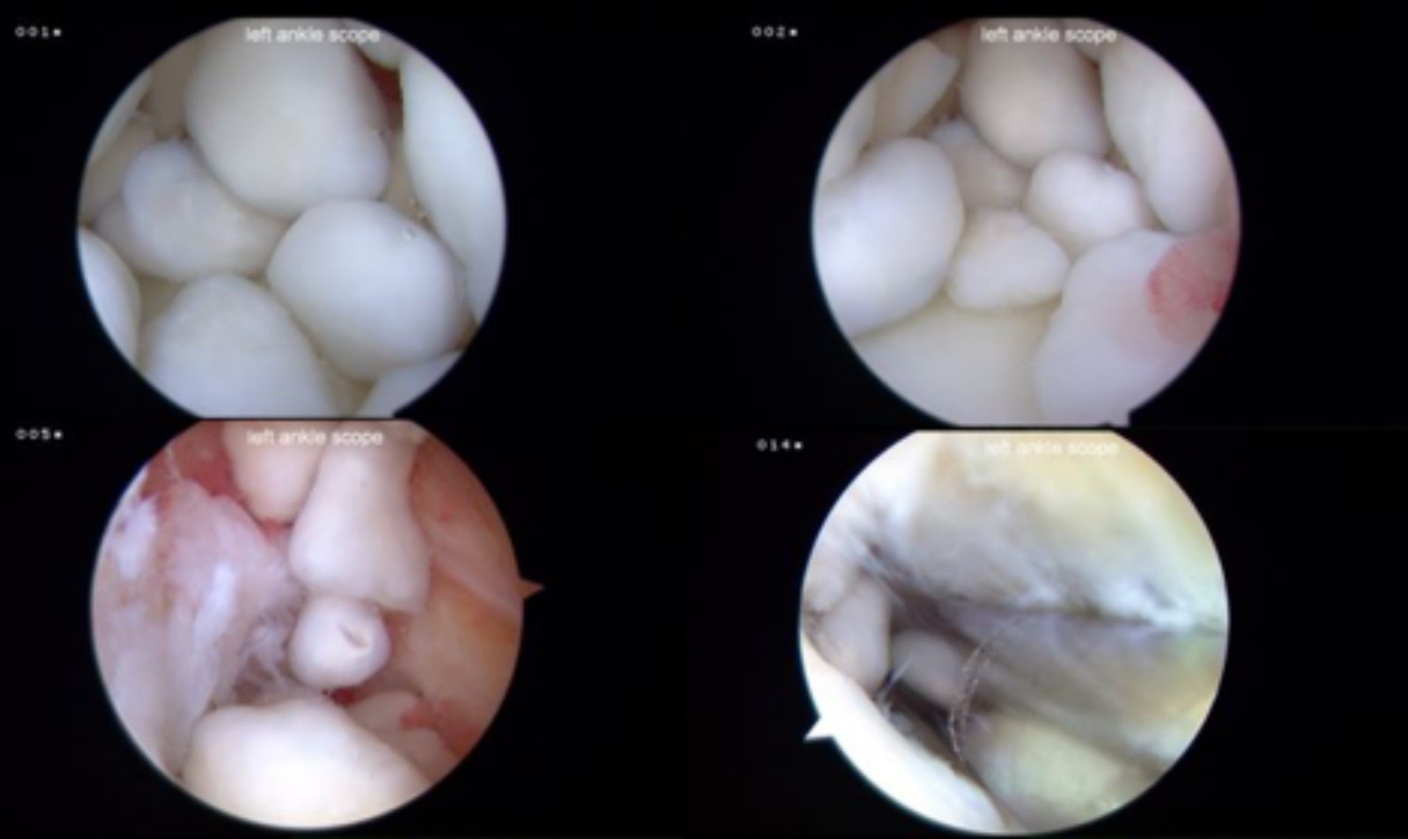
Left Ankle Joint Arthroscopic View. Left ankle joint arthroscopic view shows multiple cartilaginous loose bodies.

The patient was subsequently informed of the preliminary diagnosis of synovial chondromatosis. After the explanation of several treatment options, including continued conservative management and open loose body excision, the patient elected to undergo arthroscopic removal of the loose bodies with extensive synovectomy. The patient was taken to the operating room and placed supine on the operating table. Anteromedial and anterolateral standard portals were established using nick and spread technique after identifying the superficial peroneal nerve using surface anatomy and infiltrating the ankle joint with saline. With the ankle fully dorsiflexed, a blunt trocar was used to enter the joint followed by camera and grasper as shown in Figure 2. Visualization revealed anterior tibiotalar chondral damage, synovitis, and a plethora of cartilaginous loose bodies.

**Figure 3:**
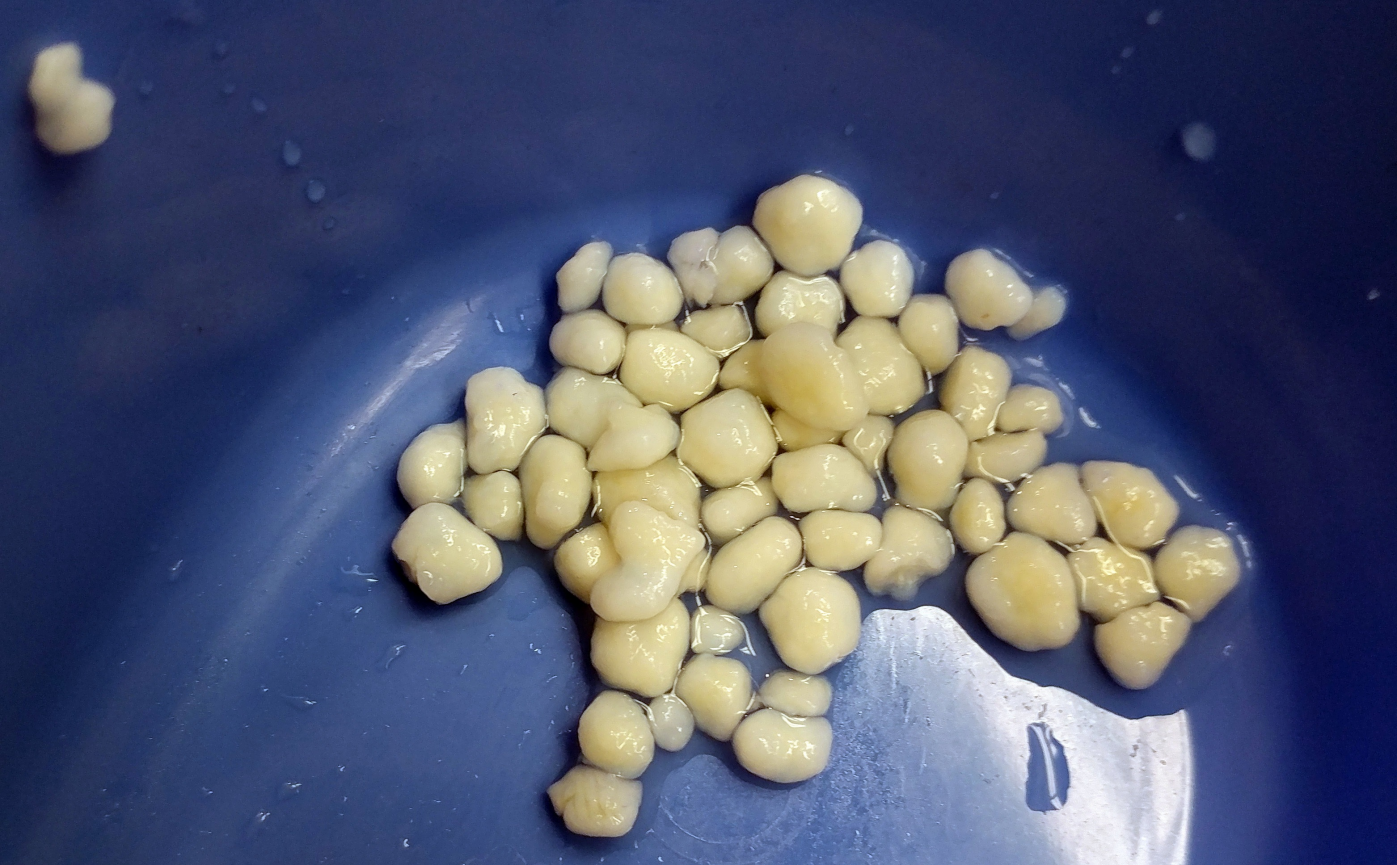
Gross Finding of Loose Bodies. Several loose bodies shown after removal from tibiotalar joint via irrigation or blunt grasper.

All aspects of the ankle joint including medial and lateral gutter were explored and loose bodies removed. A total of 76 loose bodies were identified and removed. Loose bodies were initially flushed out by the irrigation, others were captured using a blunt grasper. Many of the loose bodies removed from the joint can be seen in Figure 3, ranging from 0.3 cm up to 0.7 cm in diameter and 2.3 x 0.6 x 0.4 cm in aggregate.

**Figure 4:**
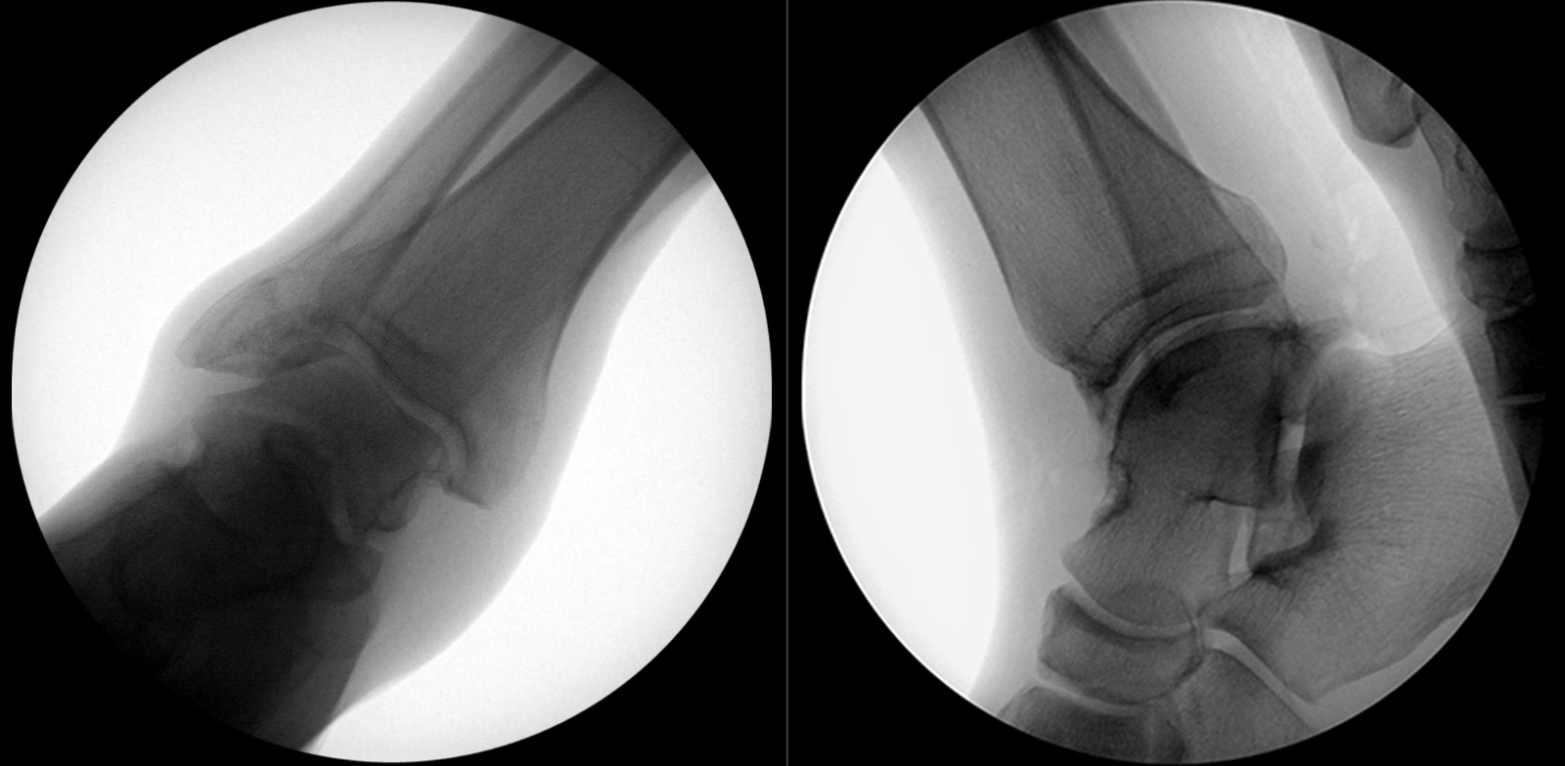
Intraoperative Fluoroscopic Imaging. Removal of all loose bodies was verified by intraoperative fluoroscopic imaging. Anteroposterior radiograph shown on the left, and lateral radiograph shown on the right.

A 3.5 mm diameter full radius shaver was used to perform synovectomy. Bone was resected to minimize anterior ankle impingement. A further portal was created at the back of the ankle joint, lateral to the Achilles tendon. The posterior aspect of the joint was then visualized to ensure no loose bodies were present there. After thorough debridement of the ankle joint and removal of all loose bodies, the joint was irrigated copiously. Intraoperative fluoroscopic imaging verified complete removal of loose bodies, as shown in Figure 4. Arthroscopic portals were closed with absorbable sutures and covered with sterile dressings. The patient tolerated the procedure well without complications. The patient was discharged from the hospital that day with limited activity restrictions to promote wound healing.

**Figure 5:**
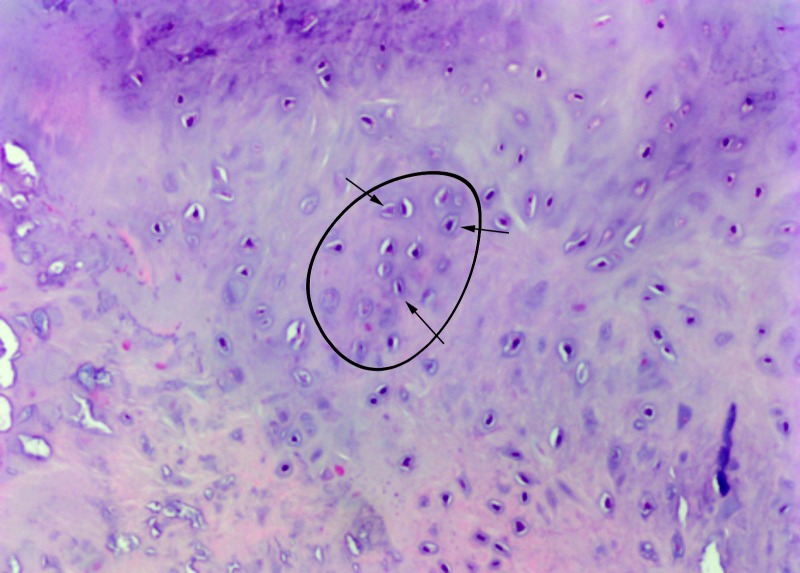
Left Ankle Joint Histology. Left ankle joint histology shows multiple well-defined nodules consisting of hyaline cartilage containing clusters of benign-appearing chondrocytes, some with degenerative atypia, consistent with synovial chondromatosis. One cluster is outlined by black ellipse. Black arrows highlight benign chondrocytes.

Following removal, the loose bodies were sent to surgical pathology. As shown in Figure 5, microscopic evaluation by the pathologist demonstrated multiple well-defined nodules made from hyaline cartilage containing clusters of benign-appearing chondrocytes, some with degenerative atypia, consistent with synovial chondromatosis.

Upon follow-up at two, six, and 12 weeks, the patient’s symptoms improved substantially. Pain and swelling decreased by the two-week follow-up appointment. By the sixth week range of motion improved to 10° of dorsiflexion and 20° of plantarflexion, the swelling was absent, and he was pain-free. The pain was rated 0 out of 10 at rest and with ambulation. Stiffness resolved with physical therapy. No other complications were encountered during follow-up care.

## Discussion

Synovial chondromatosis is a rare, mono-articular process wherein mesenchymal cells contiguous with synovial cells undergo cartilaginous metaplasia resulting in nodular formation [3]. Although usually benign, malignant transformation to chondrosarcoma has been observed and occurs in approximately 5% of cases [2, 5]. Due to the relatively rare and protracted nature of malignant transformation, there is minimal data available about incidence and prognosis of affected individuals. A recent systematic review reported malignant transformation at an average age of 56.9 years and was seen at 11.2 years from original diagnosis [6].

Patients present with symptoms that include joint pain exacerbated by activity, limited range of motion, joint effusion, and locking of the joint [7]. The target of treatment is to minimize pain, improve mechanical function, and prevent or limit progression of arthritis and chondral damage [8]. Extensive loose body removal and synovectomy have traditionally been achieved with open arthrotomy; however, we demonstrate that minimally invasive arthroscopic procedures also provide adequate treatment in synovial chondromatosis [3]. Minimally invasive surgery offers a multitude of advantages, including less pain, swelling, infection, shorter recovery times, increased patient satisfaction, and decreased morbidity [9]. To the best of the authors' knowledge, only two cases are present in the literature of synovial chondromatosis in the ankle managed arthroscopically [3, 5].

## Conclusions

We report a case of diffuse ankle synovial chondromatosis with 76 loose bodies. This case demonstrates that a three-port arthroscopic approach can provide adequate treatment while maintaining the superior risk profile inherent to arthroscopic intervention. In the future, we may consider arthroscopic approach to open arthrotomy for the treatment of synovial chondromatosis of the ankle.
